# Supplementary material for: Pox-AbDab: the orthopoxvirus antibody database
Source: Front Immunol. 2026 Jan 2;16:1698441. doi: 10.3389/fimmu.2025.1698441 (PMC12808477; doi:10.3389/fimmu.2025.1698441)
Supplement: Supplementary file 1 [file DataSheet1.pdf]

Supplementary Information
Pox-AbDab: the Orthopoxvirus Antibody Database

Henriette L. Capel[1], **Eric Ji Da Wang**[1], **Benjamin H. Williams**[1], **Charlotte M. Deane**[1 ✉], **and Matthew I. J. Raybould**[1 ✉]

[1]Oxford Protein Informatics Group, Department of Statistics, University of Oxford, 24-29 St Giles', Oxford, OX1 3LB United Kingdom

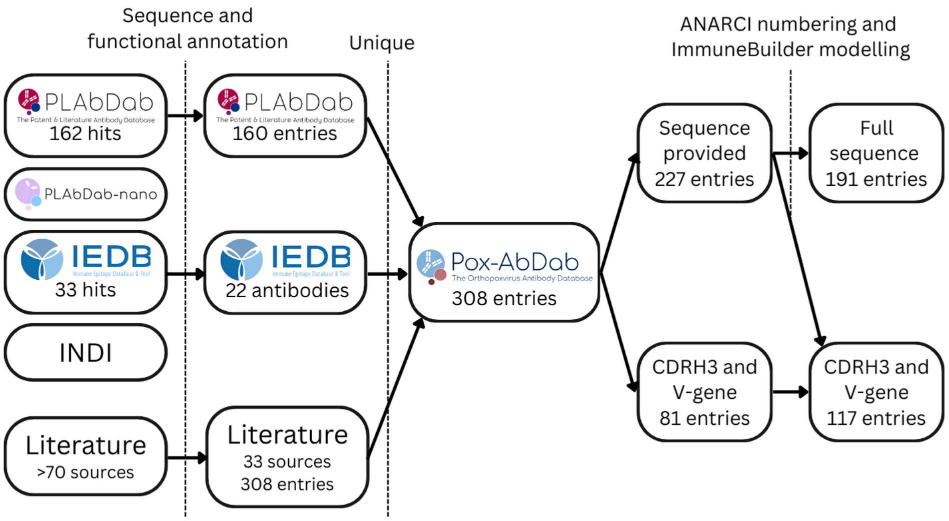

Figure S1. Data curation flow for Pox-AbDab. The publicly available databases PLAbDab (1), PLAbDab-nano (2), IEDB (3), and INDI (4) were searched for antibodies binding orthopoxvirus antigens. Additionally an exhaustive literature search on antibodies binding to orthopoxviruses was performed to collect additional antibodies for which sequence and binding/neutralisation information is available. No antibodies/nanobodies could be retrieved from PLAbDab-nano and INDI. The resulting database, Pox-AbDab contains 308 antibody entries. We filtered full sequence information based on if ANARCI (5) is able to number the sequence and ImmuneBuilder (6) is able to structurally model the sequence. This removed full sequence information for 36 antibodies as their provided sequences are incomplete.

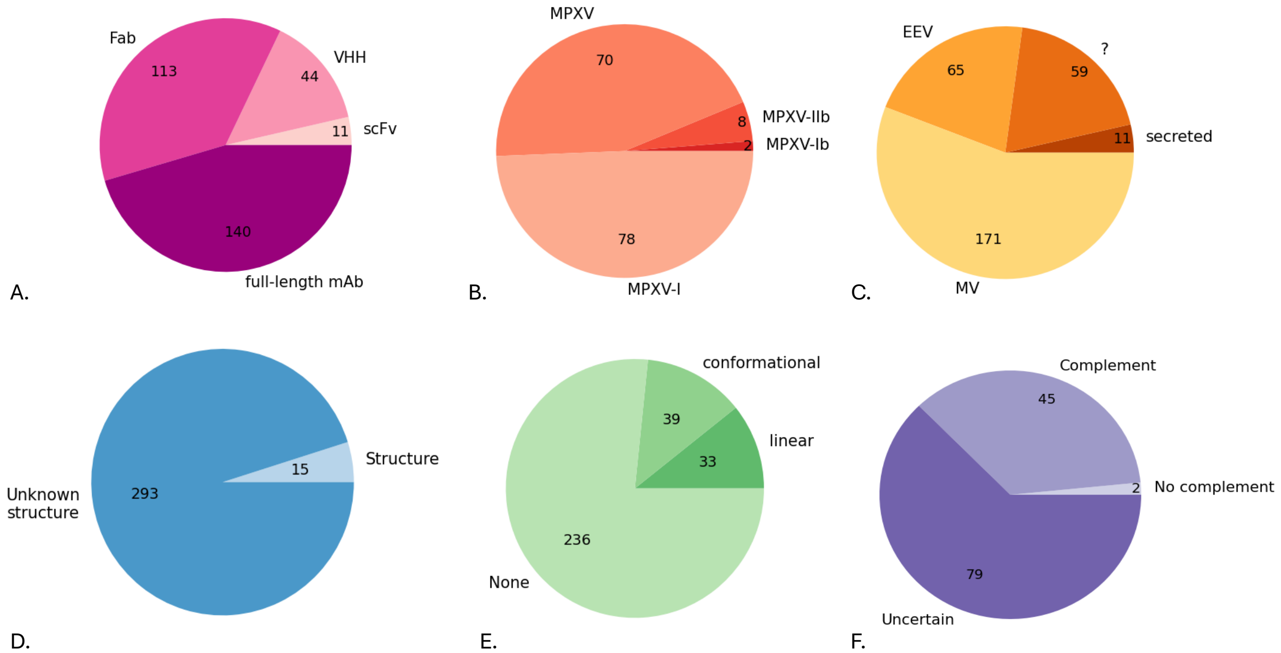

Figure S2. Pie charts showing (A) the ratios of conventional antibodies and single-domain antibodies in Pox-AbDab, with conventional antibodies split by format; (B) where a Pox-AbDab entry has been tested against MPXV, the clade that was tested (if specified, else 'MPXV'); (C) the location context of each entry, (if specified, else '?'); (D) the number of entries for which crystal structures are available; (E) the epitope information available for each Pox-AbDab entry. Linear epitope information is defined as a contiguous string of amino acids. (F) the contingency of neutralisation on an active complement system.

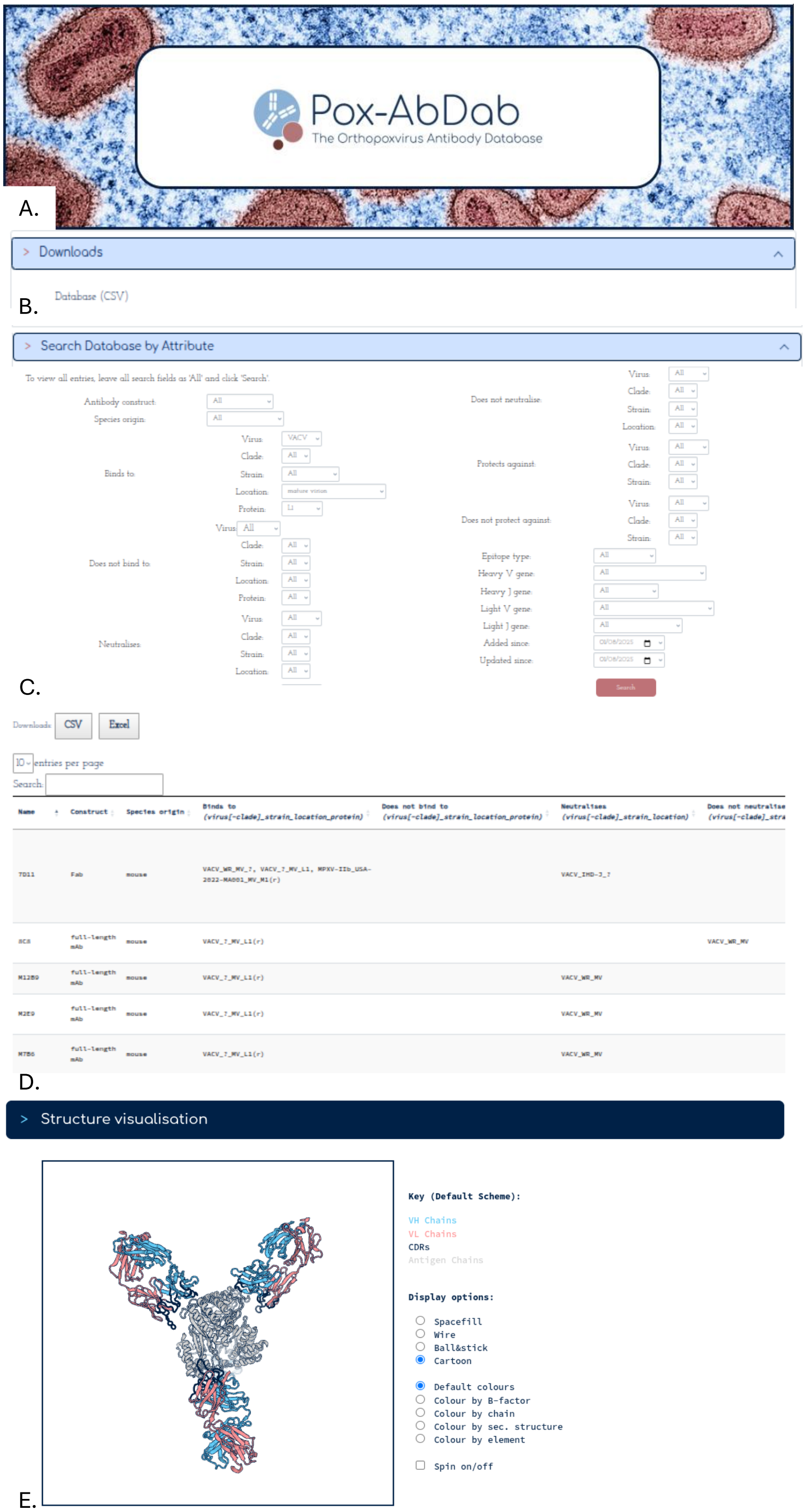

Figure S3. The Pox-AbDab Web Application. (A) The Pox-AbDab homepage logo (background image credit: NIAID, Mpox Virus, CC BY 2.0, https://www.flickr.com/photos/niaid/52988422372/in/photostream/). (B) All Pox-AbDab data can be downloaded. (C) The database can be queried by attribute (neutralisation profile, construct, germlines, etc.). (D) The result table of the attribute search. (E) The result table links the experimental solved antibody structures stored in SAbDab (7) (PDB ID 2i9l visualised here) or modelled structures by ABodyBuilder2 (6).

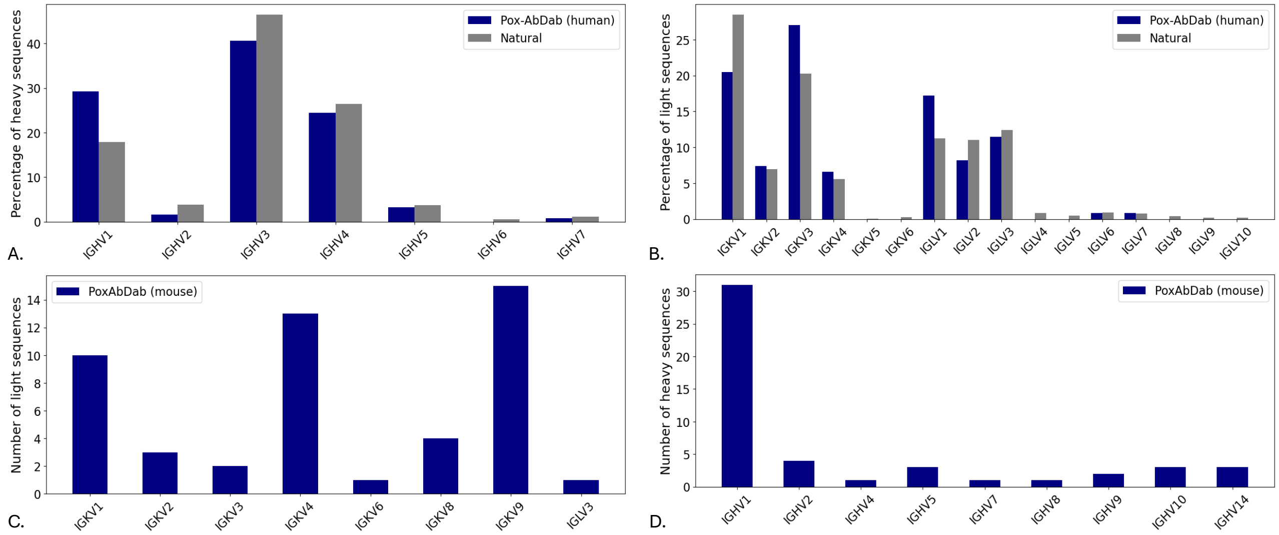

Figure S4. Bar charts showing (A) the distribution of heavy V gene family usage across the human antibodies in Pox-AbDab relative to a sample of the baseline natural repertoire from the Observed Antibody Space (OAS) database (8, 9); (B) the distribution of light V gene family usage across the human antibodies in Pox-AbDab relative to a sample of the baseline natural repertoire from the Observed Antibody Space (OAS) database (8, 9); (C) the distribution of heavy V gene family usage across the murine antibodies in Pox-AbDab; (D) the distribution of light V gene family usage across the murine antibodies in Pox-AbDab. Comparison against the natural human repertoire is based on 5000 randomly samples natural antibody sequence from the Jaffe et al., 2022 (10) as stored in OAS.

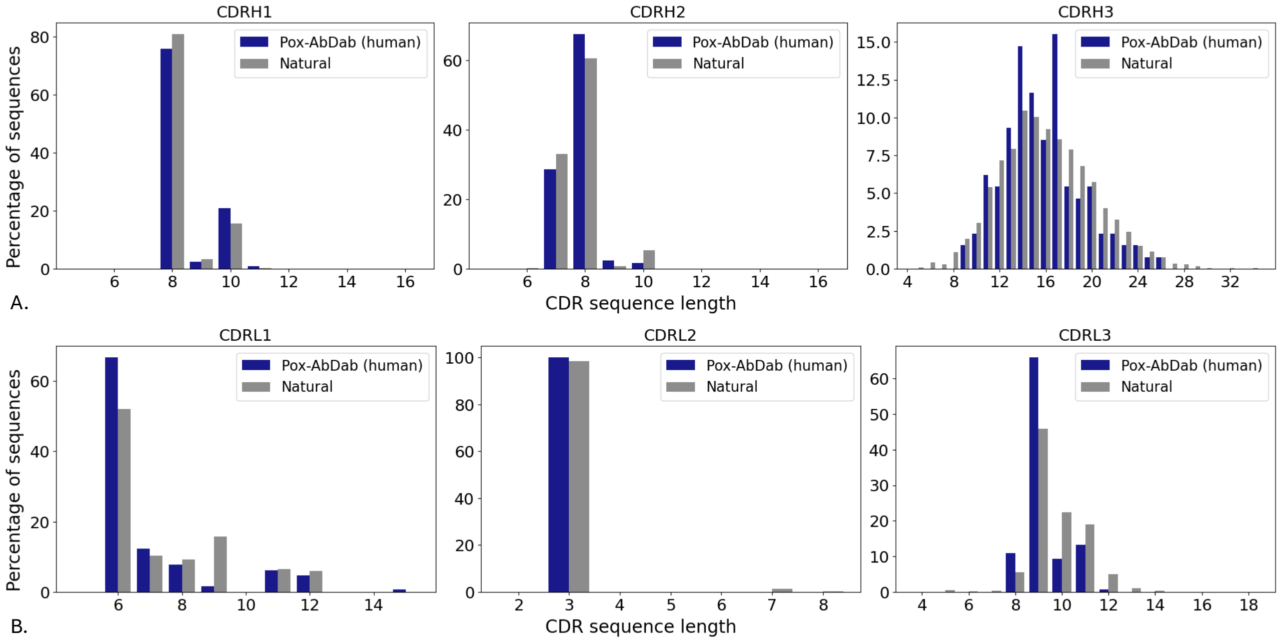

Figure S5. Bar charts showing the distributions of CDR lengths in the full-length sequenced, human antibodies in Pox-AbDab relative to a sample of the baseline natural repertoire from the Observed Antibody Space (OAS) database (8, 9): (A) across heavy chain CDRs, and (B) across light chain CDRs. Comparison against the natural human repertoire is based on 5000 randomly samples natural antibody sequence from the Jaffe et al., 2022 (10) as stored in OAS.

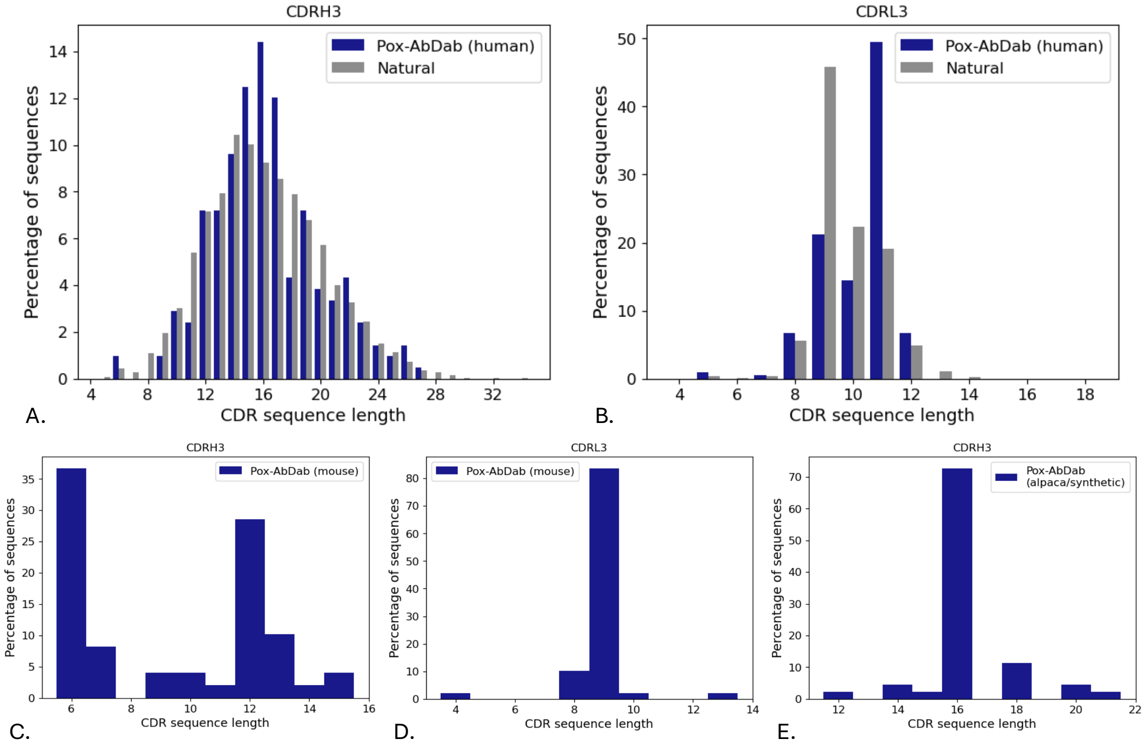

Figure S6. Bar charts showing (A) the CDRH3 and (B) the CDRL3 distributions of all the human antibodies in Pox-AbDab (including those that only have clonotype information) relative to a sample of the baseline natural repertoire from the Observed Antibody Space (OAS) database (8, 9); (C) the CDRH3 and (D) the CDRL3 distributions of all the murine antibodies in Pox-AbDab (including those that only have clonotype information); (E) the CDRH3 distribution of all single-domain antibodies in Pox-AbDab. Comparison against the natural human repertoire is based on 5000 randomly samples natural antibody sequence from the Jaffe et al., 2022 (10) as stored in OAS.

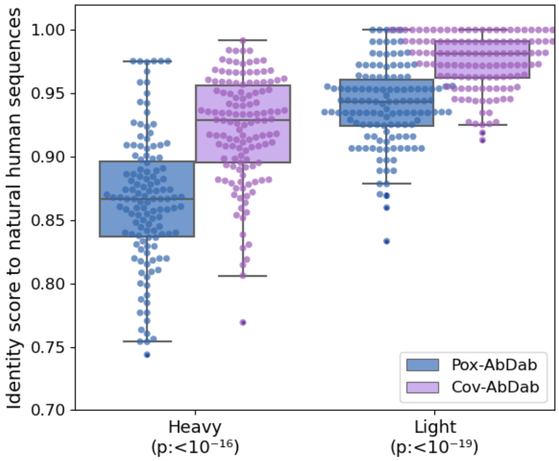

Figure S7. Antibody heavy and light sequence identity score to a set of human antibodies from naive B-cells. The 130 full-length human antibody sequences in Pox-AbDab (blue) and 130 randomly selected human antibody sequences from CoV-AbDab (purple) were compared against all human B cell receptor sequences from OAS using the "OAS-aligned" dataset provided with KA-Search (1.9Bn heavy chains from 69 studies, 350.5M light chains from 38 studies). The highest sequence identity observed was recorded. P-values of the Mann–Whitney U test are indicated on the x-axis.

Protein in VACV Nomenclature (Strain if not Copenhagen)	Location	Function	Surface protein	Number of targeting antibody sequences	Number of targeting antibody crystal structures
A10	MV	Assembly (11)	No	1	
**A13**	MV	Assembly (12)	Yes		
A14	MV	Morphogenesis (13)	Yes	5	
A16	MV	Entry/Fusion	Yes	4	
**A17**	MV	Assembly, Fusion	Yes		
A21	MV	Entry/Fusion	Yes	1	
WR148 (WR)	MV	Fusion Supressor	No	7	
**A26**	MV	Attachment	Yes		
A27	MV	Attachment	Yes	27	2
A28	MV	Entry/Fusion	Yes	1	
A33	EEV	Spread	Yes	33	3
**A34**	EEV	Spread, Attachment (14)	Yes		
**A56**	EEV	Fusion (15), Immune Evasion (16)	Yes		
B5	EEV	Spread, Morphogenesis	Yes	27	1
C3	Secreted	Immune evasion	No	11	
D8	MV	Attachment	Yes	32	5
**F5**	EEV	Plaque morphology (17)	Yes		
F9	MV	Entry/Fusion	Yes	1	
F13	EEV	Spread (18), Morphogesis (18)	Yes	1	
**G3**	MV	Entry/Fusion	Yes		
**G9**	MV	Entry/Fusion	Yes		
**H2**	MV	Entry/Fusion	Yes		
H3	MV	Assembly, Attachment	Yes	39	
H5	MV	Morphogenesis (19)	No	2	
I1	MV	Assembly (20)	No	5	
**J5**	MV	Entry/Fusion	Yes		
L1	MV	Entry/Fusion	Yes	15	2
**L5**	MV	Entry/Fusion	Yes		
**O3**	MV	Entry/Fusion	Yes		

Table S1. VACV proteins with known roles in orthopoxvirus function alongside the data availability for antibodies engaging each protein in Pox-AbDab. For the proteins highlighted in bold no antibody sequence is publicly available. All function annotations derive from Moss et al. 2011 (21), except where otherwise indicated.

Bibliography

1. Brennan Abanades, Tobias H Olsen, Matthew IJ Raybould, Broncio Aguilar-Sanjuan, Wing Ki Wong, Guy Georges, Alexander Bujotzek, and Charlotte M Deane. The Patent and Literature Antibody Database (PLAbDab): an evolving reference set of functionally diverse, literature-annotated antibody sequences and structures. *Nucleic Acids Research*, 52(D1):D545–D551, 2024.
2. Gemma L. Gordon, Alexander Greenshields-Watson, Parth Agarwal, Ashley Wong, Fergus Boyles, Alissa Hummer, Ana G. Hernandez Lujan, and Charlotte M. Deane. PLAbDab-nano: a database of camelid and shark nanobodies from patents and literature. *Nucleic Acids Research*, 53(D1):D535–D542, 2025. doi: 10.1093/nar/gkae881.
3. Randi Vita, Nina Blazeska, Daniel Marrama, IEDB Curation Team Members Shackelford Deborah Zalman Leora Foos Gabriele Zarebski Laura Chan Kenneth Reardon Brian Fitzpatrick Sidne Busse Matthew Coleman Sara Sedwick Caitlin Edwards Lindy MacFarlane Catriona Ennis Marcus, Sebastian Duesing, Jason Bennett, Jason Greenbaum, Marcus De Almeida Mendes, Jarjapu Mahita, Daniel K Wheeler, et al. The Immune Epitope Database (IEDB): 2024 update. *Nucleic Acids Research*, 53(D1):D436–D443, 2025.
4. Piotr Deszyński, Jakub Młokosiewicz, Adam Volanakis, Igor Jaszczyszyn, Natalie Castellana, Stefano Bonissone, Rajkumar Ganesan, and Konrad Krawczyk. INDI—integrated nanobody database for immunoinformatics. *Nucleic Acids Research*, 50(D1):D1273–D1281, 2022.
5. James Dunbar and Charlotte M Deane. ANARCI: antigen receptor numbering and receptor classification. *Bioinformatics*, 32(2):298–300, 2016.
6. Brennan Abanades, Wing Ki Wong, Fergus Boyles, Guy Georges, Alexander Bujotzek, and Charlotte M Deane. ImmuneBuilder: Deep-Learning models for predicting the structures of immune proteins. *Communications Biology*, 6(1):575, 2023.
7. James Dunbar, Konrad Krawczyk, Jinwoo Leem, Terry Baker, Angelika Fuchs, Guy Georges, Jiye Shi, and Charlotte M Deane. SAbDab: the structural antibody database. *Nucleic Acids Research*, 42(D1):D1140–D1146, 2014.
8. Aleksandr Kovaltsuk, Jinwoo Leem, Sebastian Kelm, James Snowden, Charlotte M. Deane, and Konrad Krawczyk. Observed Antibody Space: A Resource for Data Mining Next-Generation Sequencing of Antibody Repertoires. *Journal of Immunology*, 201(8):2502–2509, 2018. doi: 10.4049/jimmunol.1800708.
9. Tobias H Olsen, Fergus Boyles, and Charlotte M Deane. Observed Antibody Space: A diverse database of cleaned, annotated, and translated unpaired and paired antibody sequences. *Protein Science*, 31(1):141–146, 2022.
10. David B Jaffe, Payam Shahi, Bruce A Adams, Ashley M Chrisman, Peter M Finnegan, Nandhini Raman, Ariel E Royall, FuNien Tsai, Thomas Vollbrecht, Daniel S Reyes, et al. Functional antibodies exhibit light chain coherence. *Nature*, 611(7935):352–357, 2022.
11. Jiasui Liu, Simon Corroyer-Dulmont, Vojtěch Pražák, Iskander Khusainov, Karola Bahrami, Sonja Welsch, Daven Vasishtan, Agnieszka Obarska-Kosińska, Sigurdur R Thorkelsson, Kay Grünewald, et al. The palisade layer of the poxvirus core is composed of flexible A10 trimers. *Nature Structural & Molecular Biology*, 31(7):1105–1113, 2024.
12. Bethany Unger and Paula Traktman. Vaccinia virus morphogenesis: A13 phosphoprotein is required for assembly of mature virions. *Journal of Virology*, 78(16):8885–8901, 2004.
13. Paula Traktman, Ke Liu, Joseph DeMasi, Robert Rollins, Sophy Jesty, and Beth Unger. Elucidating the essential role of the A14 phosphoprotein in vaccinia virus morphogenesis: construction and characterization of a tetracycline-inducible recombinant. *Journal of Virology*, 74(8):3682–3695, 2000.
14. Stephanie R Monticelli, Amalia K Earley, Jessica Tate, and Brian M Ward. The ectodomain of the vaccinia virus glycoprotein A34 is required for cell binding by extracellular virions and contains a large region capable of interaction with glycoprotein B5. *Journal of Virology*, 93(4):10–1128, 2019.
15. Timothy R Wagenaar and Bernard Moss. Association of vaccinia virus fusion regulatory proteins with the multicomponent entry/fusion complex. *Journal of Virology*, 81(12):6286–6293, 2007.
16. Brian C DeHaven, Kushol Gupta, and Stuart N Isaacs. The vaccinia virus A56 protein: a multifunctional transmembrane glycoprotein that anchors two secreted viral proteins. *Journal of General Virology*, 92(9):1971–1980, 2011.
17. Bianca M Dobson, Dean J Procter, Natasha A Hollett, Inge EA Flesch, Timothy P Newsome, and David C Tscharke. Vaccinia virus F5 is required for normal plaque morphology in multiple cell lines but not replication in culture or virulence in mice. *Virology*, 456:145–156, 2014.
18. Peter Bryk, Matthew G Brewer, and Brian M Ward. Vaccinia virus phospholipase protein F13 promotes rapid entry of extracellular virions into cells. *Journal of Virology*, 92(11):10–1128, 2018.
19. Kathleen A Boyle, Matthew D. Greseth, and Paula Traktman. Genetic confirmation that the H5 protein is required for vaccinia virus DNA replication. *Journal of Virology*, 89(12):6312–6327, 2015.
20. Nancy Klemperer, Jeremy Ward, Elizabeth Evans, and Paula Traktman. The vaccinia virus I1 protein is essential for the assembly of mature virions. *Journal of Virology*, 71(12):9285–9294, 1997.
21. Bernard Moss. Smallpox vaccines: targets of protective immunity. *Immunological Reviews*, 239(1):8–26, 2011.
